# An Integration of MicroRNA and Transcriptome Sequencing Analysis Reveal Regulatory Roles of miRNAs in Response to Chilling Stress in Wild Rice

**DOI:** 10.3390/plants11070977

**Published:** 2022-04-03

**Authors:** Wenlong Zhao, Weiyu Xiao, Jinliang Sun, Mingxin Chen, Mingqing Ma, Yaqi Cao, Weijian Cen, Rongbai Li, Jijing Luo

**Affiliations:** 1 College of Life Science and Technology, State Key Laboratory for Conservation and Utilization of Subtropical Agro-Bioresources, Guangxi University, Nanning 530004, China; zhaowlong5@mail2.sysu.edu.cn (W.Z.); xiaoweiyu6@126.com (W.X.); sunjl1008@163.com (J.S.); cmx1462900520@126.com (M.C.); mqma@st.gxu.edu.cn (M.M.); caoyaqi8@163.com(Y.C.); cweijian@gxu.edu.cn (W.C.); 2 Agriculture College, State Key Laboratory for Conservation and Utilization of Subtropical Agro-Bioresources, Guangxi University, Nanning 530004, China; lirongbai@126.com

**Keywords:** wild rice, chilling stress, chilling tolerance locus, miRNA deep sequencing, transcriptomic profiling, target genes

## Abstract

A chromosome single segment substitution line (CSSL) DC90, which was generated by introgressing *CTS-12*, a locus derived from common wild rice (*Oryza rufipogon* Griff.), into the 9311 (*Oryza sativa* L. ssp. *indica*) background, exhibits a chilling tolerance phenotype under chilling stress. Here, an integration of microRNA (miRNA) deep sequencing and transcriptomic sequencing analysis was performed to explore the expression profiles of miRNAs and their target genes mediated by *CTS-12* under chilling stress, and to reveal the possible regulatory mechanisms of miRNAs that are involved in chilling tolerance. Integration analysis revealed that a number of differentially expressed miRNAs (DEMs) and putative target genes with different expression patterns and levels were identified in 9311 and DC90 under chilling stress. KEGG enrichment analysis revealed that the target genes that are regulated by chilling-induced miRNAs are involved in the regulation of various biological processes/pathways, including protein biosynthesis, redox process, photosynthetic process, and chloroplast development in two genotypes. CRISPR/Cas9 editing of the target genes of the key DEMs in a chilling tolerant rice variety Zhonghua 11 (ZH11) found that *LOC_Os11g48020* (*OsGL1-11*), one of the putative target genes of osa-miR1846a/b-5p and encoding a wax synthesis protein, is correlated with a chilling stress tolerance phenotype, implying osa-miR1846a/b-5p/*OsGL1-11* plays an important role in *CTS-12*-mediated chilling stress tolerance regulatory pathway(s). Therefore, we speculate that the *CTS-12* may regulate the key miRNA target genes in response to chilling stress by differential regulation of miRNAs in wild rice, thereby resulting in the variation of chilling tolerance phenotype between 9311 and DC90.

## 1. Introduction

As sessile organisms, higher plants usually adopt the ‘acclimation’ strategy upon encountering various abiotic stresses, such as chilling, heat, drought, and high salinity in their life cycles. Extreme adverse environmental conditions have a great impact on plant growth and development, and further limit crop yield and quality [1,2]. In tropical and subtropical climatic zones, many economically important crops such as rice (*Oryza sativa* L.), maize (*Zea mays* L.), and potato (*Solanum tuberosum* L.) are sensitive to chilling stress, which is a major constraint environmental factor for crop production that can cause great yield loss [2,3]. Rice is one of the staple food crops, providing carbohydrate needs for more than 50 percent of the world population. In general, the optimal temperature for rice germination and seedling growth is 25–35 °C [4,5,6,7]. The physiological metabolism of rice seedlings becomes vulnerable when the temperature falls below 15 °C [8]. Under chilling conditions, slow growth, yellowing, wilting, tillering reduction, spikelet sterility, and reduced rates of photosynthesis are observed in any rice growth stage, and even cause rice plant death under severe circumstances [9,10,11,12]. Therefore, it is of great importance to understand the key biological factors, including miRNAs, that are involved in chilling tolerance regulation in rice.

miRNAs are a class of small noncoding RNAs that are 19 to 24 nucleotides (nt) in length [5,13]. They are encoded by endogenous genes and transcribed by RNA polymerase II. Dicer-like proteins process miRNA precursors into mature miRNAs, which then mediate transcription silencing, degradation, or translation inhibition of target mRNAs at the transcriptional and/or post-transcriptional levels to regulate their expressions [14,15]. A large number of miRNAs are well-conserved among different plant species [16], and as well, it has been reported that miRNAs play important roles in plant physiological and molecular regulation processes, including growth and development, morphogenesis, protein degradation, and signal transduction, and in responding to abiotic and biotic stresses [14,15,17,18,19,20].

So far, previous studies have demonstrated that plant miRNAs and their target genes serve as major regulatory factors in a variety of unfavorable environmental stresses [20]. For example, overexpression of the two osa-miR319 family members, osa-miR319a and osa-miR319b, and downregulation of the expression of either of their target genes, *OsPCF5* and *OsPCF8* in rice, can enhance chilling tolerance after chilling acclimation [21]. Another study also showed that osa-miR319b serves as a key factor in chilling tolerance regulation in rice [22]. Likewise, microRNA156 (miR156) plays important roles in the regulation of diverse biological processes, including stress tolerance in plants. Overexpression of rice osa-miR156 leads to increased cell viability and growth rate in Arabidopsis (*Arabidopsis thaliana*), pine (*Pinus elliottii*), and rice under chilling stress [23]. In rice, osa-miR156 targets 11 SQUAMOSA promoter-binding protein-like (SPL) genes that encode plant-specific transcription factors. Specifically, osa-miR156 targets *OsSPL3* that can result in the reduction of the expression of *OsWRKY71* to regulate chilling stress tolerance under chilling conditions [23,24]. For osa-miR408, interestingly, its constitutive expression can enhance chilling tolerance but reduce drought tolerance of rice plants, suggesting that osa-miR408 plays distinct roles in responding to low temperature and drought stresses [25]. In addition, overexpression of osa-miR535 results in chilling-induced cell death, ROS overaccumulation, and osmotic regulation impairment, indicating the negative regulation of osa-miR535 in response to chilling stress in rice [26].

With the rapid development of bioinformatics and sequencing technology, high-throughput sequencing has become one of the most important approaches to inferring plant miRNA regulation processes under abiotic stresses. Meanwhile, miRNA mediated gene silencing at the transcriptional and post-transcriptional levels has attracted increasing attention. Identification of more plant-specific miRNAs and a variety of biological processes in which their target genes are involved by high-throughput sequencing will be important for unraveling pathways/mechanisms regulated by plant miRNAs [27]. Through miRNA sequencing, 16 significant differentially expressed miRNAs (9 up-/7 downregulated) were identified in Dongxiang wild rice under chilling stress [28]. Among these miRNAs, osa-miR408-5p is significantly upregulated by chilling stress. Overexpression of osa-miR408 has been shown to enhance chilling tolerance of rice [25,28]. In wheat (*Triticum aestivum* L.), 34 known and 5 novel differentially expressed miRNAs, including *miR156*, *miR159*, *miR169*, and *miR319*, were identified under chilling stress using miRNA sequencing [29]. Using the same approach, 3 known and 25 predicted differentially expressed miRNAs, including *miR169*, *miR172*, and *miR397*, were identified in chilling-treated samples in Brachypodium (*Brachypodium sylvaticum*) [30]. In addition, microarray technology has also been widely utilized to identify miRNAs in plants. For example, 19 and 10 chilling stress-responsive miRNAs were respectively identified in Populus (*Populus trichocarpa*) and Arabidopsis through microarray analysis, respectively [31,32]. These indicate that high-throughput sequencing and microarray approaches are effective ways to identify miRNAs and to infer the miRNA-mediated regulatory pathways in response to adverse environmental conditions in plants.

Prediction of the miRNA target genes is of great significance for elucidating regulatory pathways that are related to the interested biological processes. In recent years, despite an increase in the extensive studies on plant miRNAs, knowledge about the roles of miRNAs in response to chilling stress in rice remains to be elucidated. In this study, DC90, a previously developed CSSL that contains the chilling-tolerant wild rice locus *CTS-12* [33], as well as its recurrent parent 9311 (a chilling sensitive variety), were used as materials to explore the differential expression of miRNAs in response to chilling stress and to infer the possible mechanism(s) of *CTS-12*-mediated miRNAs that are involved in chilling tolerance regulations in Guangxi common wild rice.

## 2. Results

### 2.1. Identification of miRNAs That Are Involved in Chilling Stress Response via Small RNA Sequencing

DC90 exhibited a chilling-tolerance phenotype and had a high survival rate in comparison with its recurrent parent, 9311 (Figure S1a–d) [33]. Thus, DC90 was selected for miRNA sequencing to investigate the *CTS-12*-mediated miRNAs that may be involved in chilling tolerance regulations in wild rice. Here, we chose 96-h chilling-treated samples to perform miRNA and mRNA sequencings for it is the closest point preceding to the variation of the chilling stress phenotype between two genotypes in which the alterations of miRNA/transcripts in rice plants are most likely associated with the phenotypic variations and can provide informative clues for the study (unpublished data).

Small RNAs were isolated from the chilling-treated samples of 9311 and DC90 (three biological replications each) and were used to construct sequencing libraries. Small RNA sequencing generated a total of 273,795,746 raw reads (Table S1). After removal of contaminating reads, we obtained an average of 12,106,939 clean reads, and the Q30 (%) value of each library was higher than 96% (Table S1), indicating the high quality of the small RNA sequencing. The statistical analysis of the length of small RNA obtained from the sequencing showed that 21-nt miRNAs were the most abundant category in the group of known miRNAs and 24-nt were most abundant category in the group of novel miRNAs (Figures S2 and S3).

**Figure 1:**
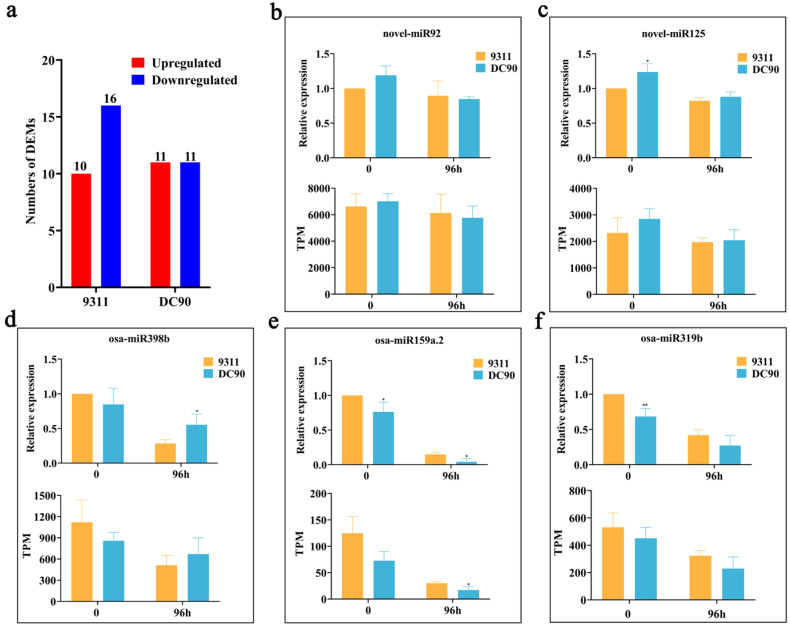
Small RNA sequencing analysis of the chilling-treated samples of 9311 and DC90. (**a**) The numbers of DEMs identified in 9311 and DC90. (**b**–**f**) Validation of the quality of miRNA-seq by qRT-PCR. U6 was used as an endogenous reference for qRT-PCR. The sequences of the primers are shown in Table S16. The data are presented as mean ± SD (n = 3). * and ** represent significant at *p* ≤ 0.05 and 0.01 levels according to Student’s *t*-test, respectively.

DEMs in the 96-h chilling-treated samples were determined by comparing the expression levels with their respective controls (0 h). False discovery rate (FDR) ≤ 0.05 and |FC (Fold Change)| ≥ 1.5 (|Log2(FC)| ≥ 0.58) were used as the cutting-off threshold. In total, 26 DEMs (10 up-/16 downregulated) were identified in 9311, and 22 DEMs (11 up-/11 downregulated) were identified in DC90 after 96-h chilling stress treatment (Figure 1a, Tables S2 and S3). To validate the quality of small RNA sequencing, five DEMs were randomly selected for qRT-PCR (Figure 1b–f). In line with the results of miRNA-seq, the trends of expression changes of these DEMs (osa-miR319b, osa-miR398b, osa-miR159a.2, novel_miR92, and novel_miR125) in the indicated time-points observed in qRT-PCR analysis were similar to those of them in small RNA sequencing, indicating that the results of miRNA-seq were reliable. 

### 2.2. Expression Profile Analysis of Chilling-Induced miRNAs

**Figure 2:**
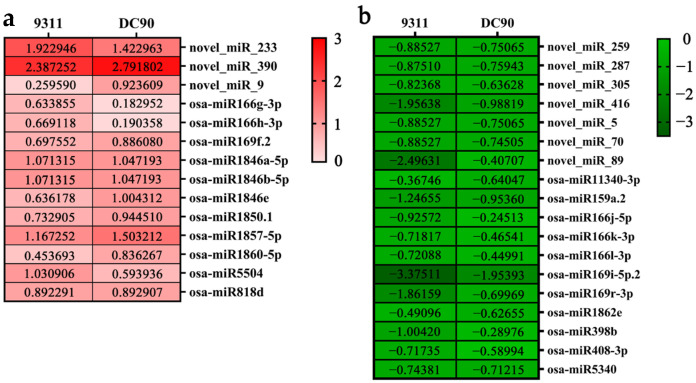
Differential expression analysis of DEMs identified in 9311 and DC90 under chilling stress conditions. (**a**,**b**) Heat map of relative expression levels of DEMs; red heat map represents upregulation and green heat map represents downregulation. Heat map data were subjected to log2 transformation of FC. FC ≥ 1.5 (|Log2(FC)| ≥ 0.58) is the cutting-off threshold for identifying significant DEMs.

The expression pattern analyses showed that the majority of the DEMs exhibited totally different expression patterns between 9311 and DC90, of which, osa-miR166h-3p and osa-miR166g-3p, two DEMs of the miR166 family, were significantly upregulated in 9311, whereas no significant changes were observed in DC90 in contrast to their non-treated controls (Figure 2a). Conversely, three other DEMs (osa-miR166j-5p, osa-miR166k-3p, and osa-miR166l-3p) of this family were significantly downregulated in 9311, and also no significant changes were observed in DC90 (Figure 2b), indicating that, although the above five miRNAs belong to the same miRNA family, they exhibited different expression patterns and may have different roles in chilling response in rice. Distinctly, as members of the miR169 family, osa-miR169i-5p.2 and osa-miR169r-3p were significantly downregulated both in 9311 and DC90; nonetheless, their downregulation levels in 9311 was lower than those of in DC90 (Figure 2b). Likewise, the expression level of osa-miR159a.2 was significantly declined in 9311 and DC90 under chilling stress, but the decline in 9311 was lower than DC90 (Figure 2b). In contrast, osa-miR398b and osa-miR408-3p were significantly downregulated only in 9311 but not in DC90 in response to chilling stress (Figure 2b). Among the identified novel miRNA, novel_miR_9 was upregulated only in DC90 but not in 9311; on the contrary, novel_miR89 was downregulated only in 9311 but not in DC90 (Figure 2a,b). Interestingly, in general, the number of downregulated DEMs in 9311 was more than those of in DC90 under chilling stress, implying that chilling stress severely inhibited the expression of miRNAs in 9311, and the downregulation of these miRNAs may result in chilling sensitivity of 9311. In addition, several DEMs had the same expression directions and had significant strong expression inductions, such as novel_miR_233 and novel_miR_390, which were significantly upregulated, and osa-miR169i-5p.2, which was significantly downregulated in two genotypes (Figure 2a,b).

### 2.3. Integration of mRNA-Seq and miRNA-Seq to Predict Target Genes of the DEMs

**Figure 3:**
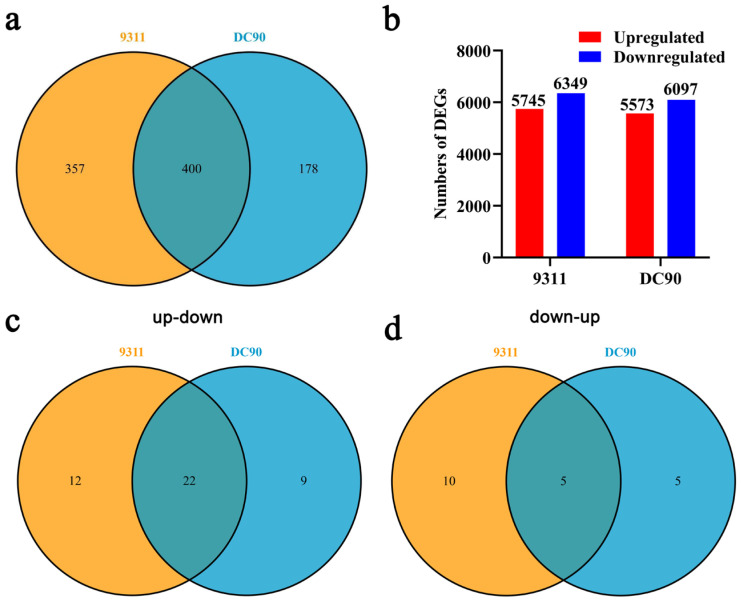
Transcriptomic profiling analysis of the chilling-treated samples of 9311 and DC90. (**a**) The numbers of putative target genes of DEMs that were identified in 9311 and DC90. (**b**) The numbers of DEGs identified in 9311 and DC90. (**c**,**d**), Venn diagram showing the numbers of target genes of DEMs identified in DC90 and 9311. up-down represents the group of down-regulated target genes that were regulated by the up-regulated group of DEMs; down-up, represents the group of up-regulated target genes that were regulated by the down-regulated group of DEMs. |log2(FC)| ≥ 1 is the cutting-off threshold for identifying significant DEGs.

Further, putative target genes of the identified DEMs were predicted using the TargetFinder program [34]. In 9311, the identified DEMs were predicted to target 757 genes, and in DC90, a total of 578 genes were predicted to be targeted by identified DEMs (Figure 1a, Table S2), among which, 400 target genes of DEMs were common between two genotypes; 357 (38.2%) target genes were specifically identified in 9311 and 178 (19.0%) target genes were specifically identified in DC90 (Figure 3a, Table S3). 

To confirm the regulatory relationship between the identified DEMs and their putative target genes under chilling stress, we performed transcriptome sequencing to investigate the expression alterations of the target genes under chilling stress. Total RNA of 96-h chilling-treated and non-treated whole plant samples (three biological replicates each) were isolated for transcriptome sequencing to identified DEGs. The significant DEGs were determined using |log2(FC)| ≥ 1 and FDR ≤ 0.01 as thresholds. In mRNA-seq, we identified 12094 and 11670 DEGs in chilling-induced 9311 and DC90 samples, respectively. Among these DEGs, 5745 up-/6349 downregulated genes were identified in 9311, while 5573 up-/6097 downregulated genes were identified in DC90 (Figure 3b, Tables S4–S7). The quality of mRNA-seq was validated by qRT-PCR. The results showed that the expression changes of six randomly selected DEGs examined in qRT-PCR analysis were similar to those of them in mRNA-seq, indicating the reliability of the sequencing results (Figure S4).

In subsequent, the regulatory relationship between DEMs and their putative significantly differentially expressed target genes (hereafter, designated as SDETGs) were preliminary determined on the basis of their expression directions. In the upregulated group of the DEMs identified in two genotypes, 43 predicted SDETGs were downregulated, of which, 22 were common between 9311 and DC90, while 12 and 9 SDETGs were specifically identified in 9311 and DC90, respectively (Figure 1a and Figure 3c). In the downregulated group of DEMs, 20 predicted SDETGs were upregulated. Among them, five were common between 9311 and DC90, and 10 and 5 of the SDETGs were specifically identified in 9311 and DC90, respectively (Figure 1a and Figure 3d).

### 2.4. Analysis of the Regulatory Relationship between DEMs and Target Genes Based on the Expression Regulatory Networks

To gain deep insight into the regulatory relationship between the identified miRNAs and their putative target genes that may be involved in chilling tolerance regulation, we built regulatory networks of DEMs and their SDETGs on the basis of the expression data using Cytoscape v3.8.2 [35]. 

**Figure 4:**
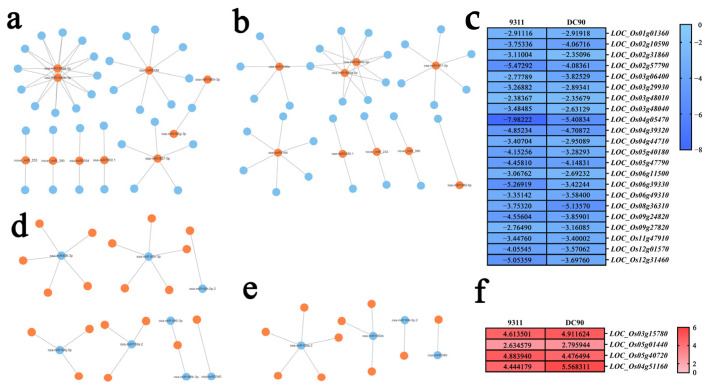
miRNAs targeted gene expression regulatory networks under chilling stress. (**a**,**d**) The regulatory networks between DEMs and SDETGs identified in 9311. (**b**,**e**) The regulatory networks between DEMs and SDETGs identified in DC90. (**c**) Expression heat map of common downregulated SDETGs that are presented in (**a**,**b**), and (**f**) common upregulated SDETGs that are presented in (**d**,**e**). Orange node represents upregulation and blue node represents downregulation. Heat map data used in the analysis were subjected to log2 transformation of FC.

In 9311, a total of 10 upregulated DEMs can be involved in building regulatory networks with predicted SDETGs, and these miRNAs target 34 genes and downregulate their expressions (Figure 1a, Figure 2a and Figure 4a, Table S8). Among these miRNAs, the expression of osa-mir166h-3p and osa-mir166g-3p was significantly elevated in 9311 (Figure 2a). Meanwhile, a total of nine upregulated DC90 DEMs and their predicted SDEGTs can be involved in building regulatory networks, and these miRNAs were predicted to target 31 genes and downregulate their expression (Figure 1a, Figure 2a and Figure 4b, Table S9); among them, osa-mir1860-5p and novel_miR_9 were significantly increased in DC90 (Figure 2a). Furthermore, seven upregulated DEMs were common between 9311 and DC90, targeting 22 genes (Figure 2a and Table S10), among which, 16 target genes were downregulated to a lower level in 9311 (Figure 4c, Table S11), suggesting that chilling stress had a greater impact on 9311. In the downregulated group of DEMs, a total of eight DEMs and predicted SDETGs, which were identified in 9311, can be involved in building regulatory networks (Figure 1a). Of which, 19 putative target genes were found to be upregulated, accompanied by the downregulation of these miRNAs (Figure 1a, Figure 2b and Figure 4d, Table S12). In DC90, a total of four DEMs can be involved in building regulatory networks, and they were predicted to target 10 SDETGs and enhanced their expressions (Figure 1a, Figure 2b and Figure 4e, Table S13). Furthermore, there were three downregulated DEMs common between 9311 and DC90 (Figure 2b, Table S14). In details, the expression levels of the three common miRNAs, including osa-miR5340, osa-mir169i-5p.2, and osa-miR159a.2, were downregulated; conversely, the expression of their predicted target genes (*LOC_Os03g15780*, *LOC_Os05g40720*, *LOC_Os04g51160*, and *LOC_Os05g01440*) was consistently upregulated under chilling stress (Figure 2b and Figure 4f, Table S15).

Overall, most target genes involved in the regulatory networks had a different extent of lower downregulated levels in 9311 than those of in DC90 (Figure 4c). 

### 2.5. KEGG Enrichment Analysis of DEM Target Genes

**Figure 5:**
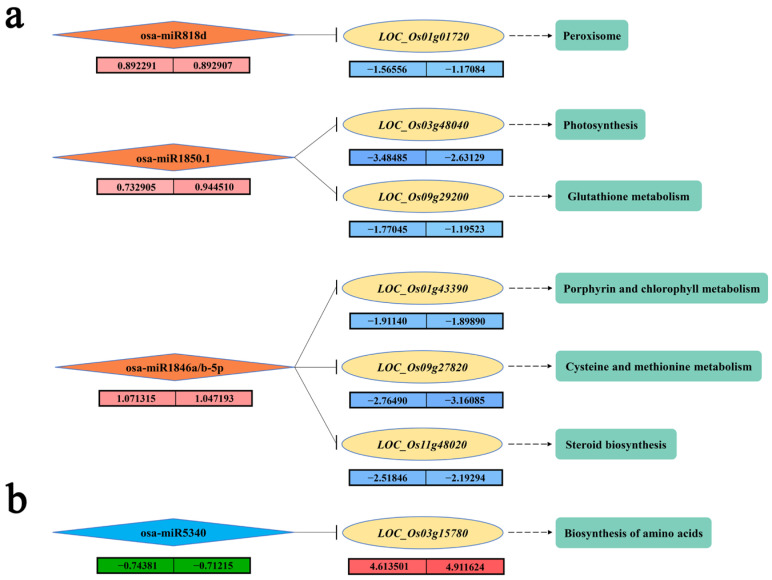
Regulatory relationship between DEMs and SDETGs. (**a**) KEGG enrichment analysis of the SDETGs of downregulated DEMs. The numbers in pink and blue boxes (9311 on the left and DC90 on the right) represent the expression levels of DEMs and SDETGs, respectively. (**b**) KEGG enrichment analysis of the SDETGs of upregulated DEMs. The numbers in green and red boxes (9311 on the left and DC90 on the right) represent the expression levels of DEMs and SDETGs, respectively.

To understand the biological functions of the target genes regulated by DEMs, KEGG enrichment analysis of the SDETGs that were common between 9311 and DC90 were performed (Figure 5a,b). For the predicted target genes of the upregulated group of DEMs, *LOC_Os01g01720*, a SDETG of osa-miR818d was downregulated to a lower level in 9311 than that of in DC90, and it was enriched in the peroxisome pathway (ko041460). Two predicted SDETGs of osa-miR1850.1, *LOC_Os03g48040* (*OsFdC2*) and *LOC_Os09g29200*, which have a similar lower expression level in 9311 in comparison with DC90, were enriched in the photosynthesis pathway (ko00195) and the glutathione metabolism pathway (ko00480), respectively. *LOC_Os03g48040*, encoding a ferredoxin, affects photosynthetic competence by controlling chloroplast development and chlorophyll content in rice [36,37]. A lower downregulation level of this gene in 9311 under chilling stress may result in severe impairing of its chloroplast development when exposed to low temperature. Moreover, *LOC_Os01g43390*, *LOC_Os09g27820*, and *LOC_Os11g48020*, three predicted SDETGs of osa-miR1846a/b-5p, were enriched in the porphyrin and chlorophyll metabolism pathway (ko00860), cysteine and methionine metabolism pathway (ko00270), and the steroid biosynthesis pathway (ko00100), respectively. *LOC_Os01g43390* and *LOC_Os11g48020* were also downregulated to a lower level in 9311 in comparison with DC90 under chilling stress, whereas *LOC_Os09g27820* was downregulated to a lower level in DC90. In addition, *LOC_Os05g40180* (*OsSTN8*), a predicted target gene of osa-miR818d, which encodes a serine/threonine protein kinase that phosphorylates photosystem II core protein and cannot be enriched in a certain KEGG term [38], was identified in the expression regulatory network and has a similar severe downregulation in 9311, suggesting that more severe damage to photosystem II occurred in 9311 under chilling stress (Figure 5a). Among the predicted target genes of the downregulated group of DEMs, *LOC_Os03g15780*, a SDETG of osa-miR5340 was the only target gene enriched in a KEGG term, the biosynthesis of the amino acids pathway (ko01230). The expression of *LOC_Os03g15780* was significantly enhanced in both 9311 and DC90 (Figure 5b). Taken together, our results suggest that chilling stress impacts on many aspects of plant biological processes, including the miRNA-mediated redox process, photosynthesis, steroid synthesis, and biosynthesis of amino acids.

### 2.6. Validation of the Regulatory Relationship between DEMs and SDETGs

**Figure 6:**
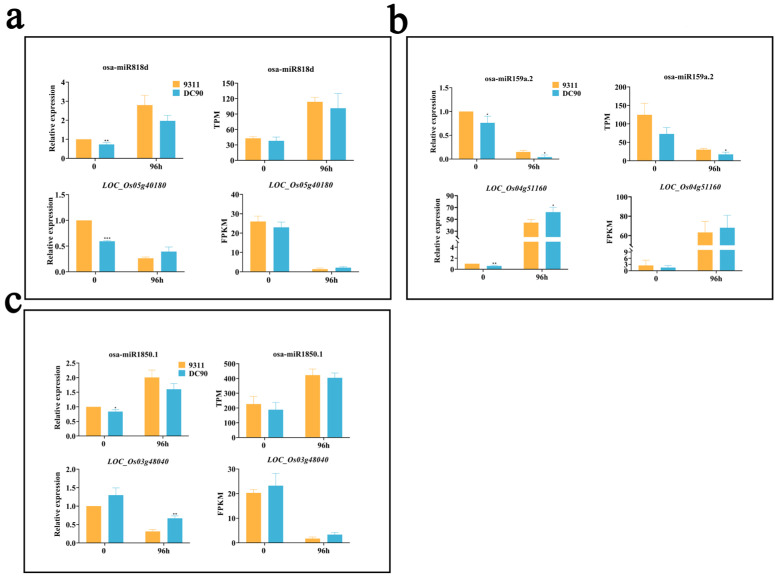
qRT-PCR validation of the expression DEMs and their SDETGs (**a**–**c**). The ACTIN gene (*Os11g0163100*) and U6 were used as endogenous references for qRT-PCR. The data are presented as mean ± SD (n = 3). *, **, and *** represent significance at *p* ≤ 0.05, 0.01, and 0.001 levels according to Student’s *t*-test, respectively.

To confirm the possible expression regulatory relationship between DEMs and their predicted SDETGs, we performed qRT-PCR to examine the expression of the target genes under chilling treatment (Figure 6a–c). The results showed that relative expression of osa-miR818d was increased in response to chilling stress in two genotypes with a higher level in 9311; correspondingly, the relative expression of its target gene *LOC_Os05g40180* was decreased under chilling stress treatment and exhibited a lower downregulation level in 9311, indicating a negative correlation between the expression profiles of osa-miR818d and *LOC_Os05g40180*. The opposite expression patterns of osa-miR1850.1 and its predicted target gene *LOC_Os09g29200* in both 9311 and DC90 were observed in miRNA-seq and RNA-seq, respectively (Figure 5a). Likewise, the expression alterations of osa-miR159a.2 and its target gene *LOC_Os04g51160* also exhibited opposite trends in the results of qRT-PCR and miRNA-seq (Figure 6b). Furthermore, the relative expression of osa-miR1850.1 was higher in 9311 than in DC90 during chilling stress, whereas its predicted target gene *LOC_Os03g48040* had a lower relative expression in 9311 in comparison with that of DC90. The similar trends were also observed in miRNA sequencing (Figure 6c).

Taken together, these results indicate that, in terms of expression profiles, a negative correlation between tested miRNAs and their predicted target genes was detected in this study, suggesting the involvement of *CTS-12*-mediated miRNA target gene regulatory processes during chilling stress response.

### 2.7. CRISPR/Cas9-Edited Putative SDETG, OsGL1-11, Leads to Chilling Sensitive Phenotype in Rice

**Figure 7:**
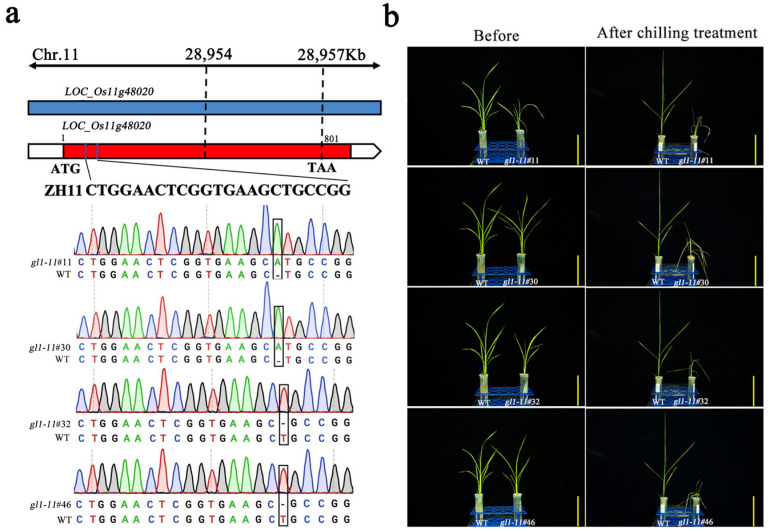
CRISPR/Cas9-edited *OsGL1-11* gene and the chilling stress phenotype of CRISPR/Cas9-edited lines. (**a**) Targeted site designed in the coding sequence of *OsGL1-11* and the edited sequences of target site in two knockout lines. (**b**) The chilling stress phenotype of *OsGL1-11* CRISPR/Cas9-edited lines. Scar bar = 5 cm. WT designates ZH11 variety.

To further investigate whether the identified putative target genes of miRNAs are correlated with the chilling tolerance phenotype of wild rice, the SDETGs of the identified DEMs including *LOC_Os11g48020* (a predicted target of osa-miR1846a/b-5), *LOC_Os03g15780* (a predicted target of osa-miR5340), and *LOC_Os03g48040* (a predicted of osa-miR1850.1) in ZH11, a *japonica* variety with high chilling tolerance, were subjected to CRISPR/Cas9 editing to preliminarily examine their chilling stress phenotype. The obtained transgenic plants were grown under normal conditions to three-leaf stage and then exposed to 8/6 °C (day/night) for 5 days with a photoperiod of day—13 h/night—11 h by supplementation with artificial light (20,000 Lux and 65% humidity) to examine chilling stress phenotype (Figure 7a,b). Among all the transgenic lines, only CRISPR/Cas9-edited *LOC_Os11g48020* lines showed chilling sensitivity in comparison with ZH11 (Figure 7b). The results suggest that not all of the chilling-induced miRNA/target gene regulatory pathways were correlated with chilling stress phenotype. *LOC_Os11g48020* (*OsGL1-11*) is one of the members of the wax synthesis gene family [39] and is involved in the steroid biosynthesis pathway. Wax forms a hydrophobic barrier on aerial plant organs. It plays an important role in protecting plants from damage caused by environmental stresses [39,40,41,42]. Therefore, our results suggest that the osa-miR1846a/b-5/*LOC_Os11g48020* (*OsGL1-11*) regulatory pathway plays important roles in chilling stress regulation in wild rice.

## 3. Discussion

9311 and DC90 share a similar genetic background, but they differ significantly in *CTS-12*-conferred chilling tolerance. Here, we explored the *CTS-12*-mediated possible mechanism(s) in which miRNAs are involved under chilling stress via miRNA sequencing and transcriptomic analyses. Our results reveal that the majority of miRNAs exhibited differential expression alterations in response to chilling stress in 9311 and DC90 (Figure 2), implying that these miRNAs may take regulatory roles under chilling stress through the differential regulation of their expression. Numbers of previous studies have demonstrated the involvement of miRNAs in abiotic/biotic stress tolerance regulation. For example, overexpression of miR408 in Arabidopsis improves chilling tolerance and enhances the antioxidant capacity of transgenic plants [43]. Likewise, overexpression of miR408 enhances photosynthesis efficiency in rice [44]. In our study, the expression of osa-miR408-3p was significantly downregulated during chilling stress in 9311 (Figure 2b), suggesting that the downregulation of osa-miR408-3p may lead to severe oxidative damage and impair photosynthetic process in rice plants under chilling stress, which in turn variates the chilling stress phenotype of 9311 and DC90. miR166, a well-conserved miRNA in plants, has an important regulatory role in responding to biotic and abiotic stresses [28,45]. It has been reported that miR166 enhances the resistance of rice to abiotic stresses by reduction of oxidative damage; moreover, miR166k and miR166h were found to positively regulate disease resistance in rice [46,47,48]. Here, the different expression extent of the members of miR166 family, including osa-miR166h-3p, osa-miR166g-3p, osa-miR166j-5p, osa-miR166k-3p, and osa-miR166l-3p, was observed in 9311 under chilling stress (Figure 2a,b), suggesting that miR166 also plays a regulatory role under chilling stress in wild rice. Moreover, miR169 family miRNAs have been reported to regulate plants in response to abiotic and biotic stresses by targeting Nuclear Factor-YA family members via the abscisic acid (ABA) pathway [49,50,51]. In this study, osa-miR169i-5p.2, osa-miR169r-3p, and osa-miR169f.2 had significant expression changes under chilling stress in both 9311 and DC90 (Figure 2a,b). Our previous study has shown that *CTS-12* mediates ABA-dependent regulation at multiple levels in response to chilling stress in wild rice [33]. Take these together, a regulatory network comprised of *CTS-12*, miRNAs, and ABA pathway in response to chilling stress in common wild rice could be proposed in our subsequent study.

In the regulatory network analysis, the chilling stress tolerance-related pathways in which DEMs and their putative target genes are involved were inferred. Four chilling responsive DEMs, osa-miR818d, osa-miR1850.1, osa-miR1846a/b-5p, and osa-miR5340, were highlighted in our results (Figure 5a,b). As a predicted SDETG of osa-miR818d, *LOC_Os01g01720* was enriched in peroxisomal pathway (ko04146). Two predicted SDETGs of osa-miR1850.1, *LOC_Os03g48040* and *LOC_Os09g29200*, were enriched in the photosynthesis pathway (ko00195) and glutathione metabolism pathway (ko00480), respectively. *LOC_Os03g48040* encodes a ferredoxin that is involved in chloroplast development in rice [36]. In addition, two predicted SDETGs of osa-miR1846a/b-5p, *LOC_Os09g27820* and *LOC_Os11g48020*, were enriched in the porphyrin and chlorophyll metabolism pathway (ko00860) and steroid biosynthesis pathway (ko00100), respectively. By contrast, osa-miR5340 was significantly downregulated both in 9311 and DC90 during chilling stress, and its predicted SDETG *LOC_Os03g15780*, which is involved in the amino acid biosynthetic pathway (ko01230), was significantly upregulated in two genotypes with a higher level in DC90. These miRNAs and their putative target genes exhibited opposite expression alteration trends during chilling stress, and the KEGG pathways were enriched, suggesting that the associated physiological processes, including redox homeostasis, chloroplast development, and photosynthesis, are differentially disturbed in two genotypes under chilling stress. Given KEGG enrichment analysis of the DEM target genes have revealed that redox homeostasis, photosynthesis, and chloroplast development pathways are involved in 9311 and DC90 chilling tolerance regulation, and the majority of the target genes of upregulated miRNA exhibited a lower expression level in 9311, our results suggest that 9311 is subjected to severe oxidative damage, as well as severe photosynthetic efficiency and chloroplast development impairment under chilling stress.

In this study, DEMs and their putative target genes were identified in the chilling-treated samples of 9311 and DC90. Some of the miRNAs/target gene regulatory pathways may correlate with chilling stress tolerance improvement. In our preliminary trials, among the CRISPR/Cas9-edited lines of the SDETGs, knockout of *LOC_Os11g48020* (*OsGL1-11*) in ZH11 background (chilling tolerance) resulted in the chilling sensitivity of transgenic lines under chilling stress (Figure 7). *LOC_Os11g48020*, one of the predicted target genes of osa-miR1846a/b-5p, is a member of the wax synthesis gene family and is involved in the steroid biosynthesis pathway [39,40,41,42]. Recently, a novel QTL *TT2* (*THEROMOTOLERANCE* 2) was identified as being associated with rice thermotolerance. *TT2*, encoding a Gγ subunit of G-protein, is associated with retention of wax under high temperature in rice. Loss of *TT2* function reduces heat-triggered Ca^2+^ signal and therefore attenuates the Ca^2+^-enhanced interaction between SCT1 (sensing Ca^2+^ transcription factor 1) and calmodulin, thereby resulting in greater wax retention and increased thermotolerance [52]. Taken together, the results suggest that *CTS*-*12* may confer chilling tolerance via mediating the osa-miR1846a/b-5/*LOC_Os11g48020* (*OsGL1-11*) regulatory pathway to regulate wax biosynthesis and retention under chilling stress in rice. 

## 4. Materials and Methods

### 4.1. Plant Materials, Growth Conditions, and Chilling Stress Treatment

The Guangxi common wild rice materials were collected by Rongbai Li and deposited in wild rice resources conservation field of Guangxi University, complying with the legislation of China. The CSSL DC90 were then developed by crossing Guangxi common wild rice DP30 with 9311 to obtain the chilling tolerance indica cultivar by Rongbai Li in our previous breeding project. Plants were grown in greenhouse conditions (13 h light at 30 °C/11 h dark at 22 °C) and in the experimental field of Guangxi University, Guangxi Province, China. The growth of rice seedlings and chilling-stress phenotype evaluation were performed according to previously described work [53]. In brief, rice seeds were grown in a plastic container with paddy soil under natural conditions with natural sunlight to three-leaf stage. Rice seedlings were then subjected to 10/8 °C (day/night) chilling treatment for 5 days with a photoperiod of day—13 h/night—11 h by supplementation with artificial light (20,000 Lux and 65% humidity) to simulate the chilling injury occurred at early rice season. The difference of chilling stress tolerance between DC90 and 9311 can be observed after 5-day chilling treatment. After treatment, the rice seedlings were allowed to recover at 28/26 °C (day/night) for 7 days, and then we scored the survival rate. During the non-lethal 96-h chilling stress treatment, rice seedling samples were collected at 0-h (untreated control) and 96-h time points. Three biological replicates of whole plant samples were collected for high-throughput deep sequencing and qRT-PCR.

### 4.2. High-Throughput Deep Sequencing of Small RNA

Total RNA of the samples of DC90 and 9311 (collected at 0- and 96-h time points) was isolated using TRIzol reagent. The small RNA libraries were constructed by using TruSeq Small RNA Sample Prep Kit (Illumina, San Diego, CA, USA) according to the manufacturer’s instructions. Small RNAs were first ligated with the 5′-RNA adaptor and then with the 3′-RNA adaptor. After first-strand synthesis and PCR amplification, the final bands were purified and submitted for sequencing on the Illumina NovaSeq6000 platform. After sequencing, the reads went through data cleaning procedures, including filtering the low-quality reads, removing reads containing unknown bases greater than 10%, filtering primer adaptor sequences, trimming adaptor contaminations, and retaining only trimmed reads of sizes from 18 to 30 nt.

The small RNA sequencing data of this study are available in Genome Sequence Archive in National Genomics Data Center, China National Center for Bioinformation/Beijing Institute of Genomics, Chinese Academy of Sciences (GSA: CRA005305).

### 4.3. Transcriptomic Profiling

Sequencing libraries were constructed using the NEBNext UltraTM RNA Library Prep Kit for Illumina according to the manufacturer’s instructions. PCR was performed with size-selected, adaptor-ligated cDNA using Phusion High-Fidelity DNA polymerase, and the products were purified (AMPure XP system). The sequencing was performed on an Illumina 2500 platform, and paired-end reads were generated.

The clean reads were mapped to rice reference genome (Rice Genome Annotation Project Release 7) using Hisat2 tools [54]. The significant differentially expressed genes (DEGs) were determined using DESeq2 [55]. |log2(FC)| ≥ 1 and FDR ≤ 0.01 were used as thresholds to identify chilling-induced DEGs.

The mRNA sequencing data of this study are available in Genome Sequence Archive in National Genomics Data Center, China National Center for Bioinformation/Beijing Institute of Genomics, Chinese Academy of Sciences (GSA: CRA005306).

### 4.4. Bioinformatics Analysis of Small RNA Sequencing Data

Clean reads were used to identify small RNAs by Bowtie software [56]. After eliminating reads belonging to rRNAs, tRNAs, snRNAs, and snoRNAs, the remaining sequences were aligned to miRBase (https://mirbase.org/ (accessed on 28 June 2021)) [57] to identify known miRNAs. In addition, the rest sequences, which can be mapped to rice reference genome, were predicted as novel miRNAs by evaluating the information of precursors in the genome locations and their structures using miRDeep2 tools [58].

The expression levels of miRNAs in each sample was normalized by transcripts per million (TPM) algorithm for comparing the expression levels of small RNAs [59]. The significant DEMs were determined using DESeq2 with the application of FC ≥ 1.5 (|Log2(FC)| ≥ 0.58) and FDR ≤ 0.05 as thresholds to identify chilling-induced DEMs. Target genes of miRNAs were predicted by TargetFinder program [34], and then compared the target gene sequences with NR, Swiss-Prot, GO, COG, KEGG, KOG, and Pfam databases using BLAST tools.

In the KEGG (Kyoto Encyclopedia of Genes and Genomes) enrichment analysis, KOBAS 3.0 (http://kobas.cbi.pku.edu.cn/kobas3 (accessed on 29 June 2021)) was used to enrich the target genes of DEMs to KEGG pathways using the *p*-value ≤ 0.05 as a cutting-off threshold. The resulting miRNAs and the predicted target genes were used for building regulatory networks, and the networks were visualized using Cytoscape v3.8.2 [35]. 

### 4.5. Quantification of miRNA Expression Level by qRT-PCR

qRT-PCR was performed to determine the quality of high-throughput sequencing of small RNA. Total RNA was isolated using TRIzol reagent according to the manufacturer’s instructions. cDNA synthesis was performed by reverse transcription (RT) with the Vazyme miRNA 1st Strand cDNA Synthesis Kit (by stem-loop) (Vazyme, Cat #MR101, Nanjing, China) according to the manufacturer’s instructions. The miRNA-specific forward primer for each miRNA and the universal reverse primer were designed by Vazyme miRNA Design V1.01 (http://www.vazyme.com/companyfile/7/ (accessed on 26 July 2021)). qPCR was performed using a Roche LightCycler 480 Real-Time PCR System in 10 μL reactions with the miRNA Universal SYBR qPCR Master Mix (Vazyme, Cat #MQ101, Nanjing, China) to detect the relative expression of these miRNAs according to the manufacturer’s instructions. The cDNA products were normalized using U6 snRNA as the endogenous reference. All of the reactions were performed in triplicate, including non-template controls. Statistical analysis was performed according to the 2^−^^ΔΔct^ method [60].

### 4.6. Generation of CRISPR/Cas9 Knockout Lines

CRISPR/Cas9-edited transgenic rice materials were purchased from Baige Biotechnology company (Hangzhou, China). For the method of generation of transgenic rice materials, in brief, the plasmids were constructed as described previously [61]. The resulting constructs with specific target sites were introduced into the calli of ZH11, a chilling tolerant *Japonica* variety with a different genetic background relative to 9311, via *Agrobacterium tumefaciens* EHA105-mediated transformation, and calli were transferred to regeneration media to obtain green plants. The T_2_ generation of homozygous knockout lines were selected for further phenotypic analysis.

## 5. Conclusions

Taken these together, we infer that *CTS-12* mediates the differential expression alterations of miRNAs and in turn differentially regulates the expression levels of their target genes in 9311 and DC90 under chilling stress. The key target genes of the identified miRNAs, which are involved in redox homeostasis, chloroplast development, and photosynthesis, are the targets for chilling responsive regulation under chilling stress, and thereby confer chilling tolerance in common wild rice.

## Data Availability

The raw sequence data reported in this paper have been deposited in the Genome Sequence Archive in National Genomics Data Center, China National Center for Bioinformation/Beijing Institute of Genomics, Chinese Academy of Sciences (GSA: CRA005305) and (GSA: CRA005306).

## Supplementary Materials

The following supporting information can be downloaded at https://www.mdpi.com/article/10.3390/plants11070977/s1, Figure S1: Chilling stress phenotypes of DC90 and 9311 at the seedling stage. (a) DC90 and 9311 seedlings before chilling stress treatment; (b) after 5-day chilling treatment; (c) and after 7-day recovery treatment. (d) Survival rate of 9311 and DC90 7-day recovery. Scale bars = 5 cm in A, B, and C. Figure S2: Length distribution of known miRNAs. Figure S3: Length distribution of novel miRNAs. Figure S4: Validation of the quality of RNA-seq data by qRT-PCR. *LOC_Os01g14440* (a), *LOC_Os01g18240* (b), *LOC_Os02g01340* (c), *LOC_Os03g59460* (d), *LOC_Os07g39480* (e), and *LOC_Os12g02450* (f) were selected for qRT-PCR. The data are presented as mean ± SD (n = 3). *, **, and *** represent significant at *p* ≤ 0.05, 0.01, and 0.001 levels according to Student’s *t*-tests, respectively. The ACTIN gene (*Os11g0163100*) was used as an endogenous reference. Table S1: Statistical analysis of sequencing data. Table S2: Differentially expressed miRNAs (DEMs) identified in the comparison of 9311-0 h vs. 9311-96 h (FC ≥ 1.5 (|Log2(FC)| ≥ 0.58) and FDR ≤ 0.05). Table S3: Differentially expressed miRNAs (DEMs) identified in the comparison of DC90-0 vs. DC90-96 (FC ≥ 1.5 (|Log2(FC)| ≥ 0.58) and FDR ≤0.05). Table S4: Upregulated differentially expressed genes (DEGs) identified in the comparison of 9311-0 h vs. 9311-96 h (|log2(FC)| ≥ 1 and FDR ≤ 0.01). Table S5: Downregulated differentially expressed genes (DEGs) identified in the comparison of 9311-0 h vs. 9311-96 h (|log2(FC)| ≥ 1 and FDR ≤ 0.01). Table S6: Upregulated differentially expressed genes (DEGs) identified in the comparison of DC90-0 h vs. DC90-96 h (|log2(FC)| ≥ 1 and FDR ≤ 0.01). Table S7: Downregulated differentially expressed genes (DEGs) identified in the comparison of DC90-0 h vs. DC90-96 h (|log2(FC)| ≥ 1 and FDR ≤ 0.01). Table S8: Regulatory relationship among upregulated DEMs and SDETGs in 9311. Table S9: Regulatory relationship among upregulated DEMs and SDETG in DC90. Table S10: List of the common upregulated DEMs and common SDETGs in 9311 and DC90. Table S11: Expression levels of common downregulated SDETGs of DEMs common between 9311 and DC90. Table S12: Regulatory relationship among downregulated DEMs and SDETG in 9311. Table S13: Regulatory relationship among downregulated DEMs and SDETG in DC90. Table S14: List of the common downregulated DEMs and common SDETGs in 9311 and DC90. Table S15: Expression levels of common upregulated SDETGs of DEMs common between 9311 and DC90. Table S16: Sequences of primers used in this study. Table S17: Summary of miRNAs and target genes identified in miRNA and mRNA sequencing.

## References

[1] Zhu J.K. (2016). Abiotic Stress Signaling and Responses in Plants. Cell.

[2] Pareek A., Khurana A., Sharma A.K., Kumar R. (2017). An overview of signaling regulons during cold stress tolerance in plants. Curr. Genom..

[3] Solanke A.U., Sharma A.K. (2008). Signal transduction during cold stress in plants. Physiol. Mol. Biol. Plants.

[4] Pradhan S.K., Pandit E., Nayak D.K., Behera L., Mohapatra T. (2019). Genes, pathways and transcription factors involved in seedling stage chilling stress tolerance in indica rice through RNA-Seq analysis. BMC Plant Biol..

[5] Xia H., Yu S., Kong D., Xiong J., Ma X., Chen L., Luo L. (2020). Temporal responses of conserved miRNAs to drought and their associations with drought tolerance and productivity in rice. BMC Genom..

[6] Pandit E., Tasleem S., Barik S.R., Mohanty D.P., Nayak D.K., Mohanty S.P., Das S., Pradhan S.K. (2017). Genome-wide association mapping reveals multiple QTLs governing tolerance response for seedling stage chilling stress in Indica Rice. Front Plant Sci..

[7] Kayess M.O., Hassan M.M., Nurhasan M., Ahmed K. (2020). Effect of low temperature on chlorophyll and carotenoid content on the seedlings of some selected boro rice varieties. Am. J. Plant Sci..

[8] Fujino K., Sekiguchi H., Sato T., Kiuchi H., Nonoue Y., Takeuchi Y., Ando T., Lin S.Y., Yano M. (2004). Mapping of quantitative trait loci controlling low-temperature germinability in rice (*Oryza sativa* L.). Theor. Appl. Genet..

[9] Andaya V.C., Mackill D.J. (2003). Mapping of QTLs associated with cold tolerance during the vegetative stage in rice. J. Exp. Bot..

[10] Suh J.P., Jeung J.U., Lee J.I., Choi Y.H., Yea J.D., Virk P.S., Mackill D.J., Jena K.K. (2010). Identification and analysis of QTLs controlling cold tolerance at the reproductive stage and validation of effective QTLs in cold-tolerant genotypes of rice (*Oryza sativa* L.). Theor. Appl. Genet..

[11] Andaya V.C., Tai T.H. (2006). Fine mapping of the *qCTS12* locus, a major QTL for seedling cold tolerance in rice. Theor. Appl. Genet..

[12] Dasgupta P., Das A., Datta S., Banerjee I., Tripathy S., Chaudhuri S. (2020). Understanding the early cold response mechanism in IR64 *indica* rice variety through comparative transcriptome analysis. BMC Genom..

[13] Voinnet O. (2009). Origin, biogenesis, and activity of plant microRNAs. Cell.

[14] Sun G. (2011). MicroRNAs and their diverse functions in plants. Plant Mol. Biol..

[15] Gielen H., Remans T., Vangronsveld J., Cuypers A. (2012). MicroRNAs in metal stress: Specific roles or secondary responses?. Int. J. Mol. Sci..

[16] Mishra R., Mohapatra R., Mahanty B., Joshi R.K. (2019). Analysis of microRNAs and their targets from onion (Allium cepa) using genome survey sequences (GSS) and expressed sequence tags (ESTs). Bioinformation.

[17] Leung A.K., Sharp P.A. (2010). MicroRNA functions in stress responses. Mol. Cell.

[18] Li C., Zhang B. (2016). MicroRNAs in control of plant development. J. Cell. Physiol..

[19] Khraiwesh B., Zhu J.K., Zhu J. (2012). Role of miRNAs and siRNAs in biotic and abiotic stress responses of plants. Biochim. Biophys. Acta.

[20] Fang Y., Xie K., Xiong L. (2014). Conserved miR164-targeted NAC genes negatively regulate drought resistance in rice. J. Exp. Bot..

[21] Yang C., Li D., Mao D., Liu X.U.E., Ji C., Li X., Zhao X., Cheng Z., Chen C., Zhu L. (2013). Overexpression of microRNA319 impacts leaf morphogenesis and leads to enhanced cold tolerance in rice (*Oryza sativa* L.). Plant Cell Environ..

[22] Unver T., Wang S.-t., Sun X.-l., Hoshino Y., Yu Y., Jia B., Sun Z.-w., Sun M.-z., Duan X.-b., Zhu Y.-m. (2014). MicroRNA319 positively regulates cold tolerance by targeting *OsPCF6* and *OsTCP21* in rice (*Oryza sativa* L.). PLoS ONE.

[23] Zhou M., Tang W. (2019). MicroRNA156 amplifies transcription factor-associated cold stress tolerance in plant cells. Mol. Genet. Genom..

[24] Cui N., Sun X., Sun M., Jia B., Duanmu H., Lv D., Duan X., Zhu Y. (2015). Overexpression of OsmiR156k leads to reduced tolerance to cold stress in rice (*Oryza Sativa*). Mol. Breed..

[25] Sun M., Yang J., Cai X., Shen Y., Cui N., Zhu Y., Jia B., Sun X. (2018). The opposite roles of OsmiR408 in cold and drought stress responses in *Oryza sativa*. Mol. Breed..

[26] Sun M., Shen Y., Yang J., Cai X., Li H., Zhu Y., Jia B., Sun X. (2020). miR535 negatively regulates cold tolerance in rice. Mol. Breed..

[27] Sunkar R., Zhou X., Zheng Y., Zhang W., Zhu J.K. (2008). Identification of novel and candidate miRNAs in rice by high throughput sequencing. BMC Plant Biol..

[28] Jiang W., Shi W., Ma X., Zhao J., Wang S., Tan L., Sun C., Liu F. (2019). Identification of microRNAs responding to cold stress in Dongxiang common wild rice. Genome.

[29] Song G., Zhang R., Zhang S., Li Y., Gao J., Han X., Chen M., Wang J., Li W., Li G. (2017). Response of microRNAs to cold treatment in the young spikes of common wheat. BMC Genom..

[30] Zhang J., Xu Y., Huan Q., Chong K. (2009). Deep sequencing of Brachypodium small RNAs at the global genome level identifies microRNAs involved in cold stress response. BMC Genom..

[31] Lu S., Sun Y.H., Chiang V.L. (2008). Stress-responsive microRNAs in Populus. Plant J..

[32] Liu H.H., Tian X., Li Y.J., Wu C.A., Zheng C.C. (2008). Microarray-based analysis of stress-regulated microRNAs in Arabidopsis thaliana. RNA.

[33] Cen W., Zhao W., Ma M., Lu S., Liu J., Cao Y., Zeng Z., Wei H., Wang S., Li R. (2020). The wild rice locus *CTS-12* mediates ABA-dependent stomatal opening modulation to limit water loss under severe chilling stress. Front. Plant Sci..

[34] Allen E., Xie Z., Gustafson A.M., Carrington J.C. (2005). microRNA-directed phasing during trans-acting siRNA biogenesis in plants. Cell.

[35] Shannon P., Markiel A., Ozier O., Baliga N.S., Wang J.T., Ramage D., Amin N., Schwikowski B., Ideker T. (2003). Cytoscape: A software environment for integrated models of biomolecular interaction networks. Genome. Res..

[36] He L., Li M., Qiu Z., Chen D., Zhang G., Wang X., Chen G., Hu J., Gao Z., Dong G. (2020). Primary leaf-type ferredoxin 1 participates in photosynthetic electron transport and carbon assimilation in rice. Plant J..

[37] Li C., Hu Y., Huang R., Ma X., Wang Y., Liao T., Zhong P., Xiao F., Sun C., Xu Z. (2015). Mutation of *FdC2* gene encoding a ferredoxin-like protein with C-terminal extension causes yellow-green leaf phenotype in rice. Plant Sci..

[38] Nath K., Poudyal R.S., Eom J.S., Park Y.S., Zulfugarov I.S., Mishra S.R., Tovuu A., Ryoo N., Yoon H.S., Nam H.G. (2013). Loss-of-function of *OsSTN8* suppresses the photosystem II core protein phosphorylation and interferes with the photosystem II repair mechanism in rice (Oryza sativa). Plant J..

[39] Islam M.A., Du H., Ning J., Ye H., Xiong L. (2009). Characterization of *Glossy1-homologous* genes in rice involved in leaf wax accumulation and drought resistance. Plant Mol. Biol..

[40] Zhou L., Ni E., Yang J., Zhou H., Liang H., Li J., Jiang D., Wang Z., Liu Z., Zhuang C. (2013). Rice *OsGL1-6* is involved in leaf cuticular wax accumulation and drought resistance. PLoS ONE.

[41] Kurokawa Y., Nagai K., Huan P.D., Shimazaki K., Qu H., Mori Y., Toda Y., Kuroha T., Hayashi N., Aiga S. (2018). Rice leaf hydrophobicity and gas films are conferred by a wax synthesis gene (*LGF1*) and contribute to flood tolerance. New Phytol..

[42] Qin B.X., Tang D., Huang J., Li M., Wu X.R., Lu L.L., Wang K.J., Yu H.X., Chen J.M., Gu M.H. (2011). Rice *OsGL1-1* is involved in leaf cuticular wax and cuticle membrane. Mol. Plant.

[43] Ma C., Burd S., Lers A. (2015). miR408 is involved in abiotic stress responses in Arabidopsis. Plant J..

[44] Pan J., Huang D., Guo Z., Kuang Z., Zhang H., Xie X., Ma Z., Gao S., Lerdau M.T., Chu C. (2018). Overexpression of microRNA408 enhances photosynthesis, growth, and seed yield in diverse plants. J. Integr. Plant Biol..

[45] Axtell M.J., Bowman J.L. (2008). Evolution of plant microRNAs and their targets. Trends Plant Sci..

[46] Salvador-Guirao R., Hsing Y.I., San Segundo B. (2018). The polycistronic miR166k-166h positively regulates rice immunity via post-transcriptional control of *EIN2*. Front. Plant Sci..

[47] Ding Y., Gong S., Wang Y., Wang F., Bao H., Sun J., Cai C., Yi K., Chen Z., Zhu C. (2018). MicroRNA166 modulates cadmium tolerance and accumulation in Rice. Plant Physiol..

[48] Zhang J., Zhang H., Srivastava A.K., Pan Y., Bai J., Fang J., Shi H., Zhu J.K. (2018). Knockdown of rice MicroRNA166 confers drought resistance by causing leaf rolling and altering stem xylem development. Plant Physiol..

[49] Zhao B., Ge L., Liang R., Li W., Ruan K., Lin H., Jin Y. (2009). Members of miR-169 family are induced by high salinity and transiently inhibit the NF-YA transcription factor. BMC Mol. Biol..

[50] Ding Q., Zeng J., He X.-Q. (2016). MiR169 and its target PagHAP2-6 regulated by ABA are involved in poplar cambium dormancy. J. Plant Physiol..

[51] Rao S., Balyan S., Jha S., Mathur S. (2020). Novel insights into expansion and functional diversification of MIR169 family in tomato. Planta.

[52] Kan Y., Mu X.-R., Zhang H., Gao J., Shan J.-X., Ye W.-W., Lin H.-X. (2022). *TT2* controls rice thermotolerance through SCT1-dependent alteration of wax biosynthesis. Nat. Plants.

[53] Cen W., Liu J., Lu S., Jia P., Yu K., Han Y., Li R., Luo J. (2018). Comparative proteomic analysis of QTL *CTS-12* derived from wild rice (Oryza rufipogon Griff.), in the regulation of cold acclimation and de-acclimation of rice (*Oryza sativa* L.) in response to severe chilling stress. BMC Plant Biol..

[54] Kim D., Langmead B., Salzberg S.L. (2015). HISAT: A fast spliced aligner with low memory requirements. Nat. Methods.

[55] Love M.I., Huber W., Anders S. (2014). Moderated estimation of fold change and dispersion for RNA-seq data with DESeq2. Genome Biol..

[56] Langmead B., Trapnell C., Pop M., Salzberg S.L. (2009). Ultrafast and memory-efficient alignment of short DNA sequences to the human genome. Genome Biol..

[57] Kozomara A., Griffiths-Jones S. (2014). miRBase: Annotating high confidence microRNAs using deep sequencing data. Nucleic Acids Res..

[58] Zhang Z., Jiang L., Wang J., Gu P., Chen M. (2015). MTide: An integrated tool for the identification of miRNA-target interaction in plants. Bioinformatics.

[59] Li B., Ruotti V., Stewart R.M., Thomson J.A., Dewey C.N. (2010). RNA-Seq gene expression estimation with read mapping uncertainty. Bioinformatics.

[60] Livak K.J., Schmittgen T.D. (2001). Analysis of relative gene expression data using real-time quantitative PCR and the 2(-Delta Delta C(T)) Method. Methods.

[61] Ma X., Zhang Q., Zhu Q., Liu W., Chen Y., Qiu R., Wang B., Yang Z., Li H., Lin Y. (2015). A robust CRISPR/Cas9 system for convenient, high-efficiency multiplex genome editing in monocot and dicot Plants. Mol. Plant.

