# Supplementary material for: An Integration of MicroRNA and Transcriptome Sequencing Analysis Reveal Regulatory Roles of miRNAs in Response to Chilling Stress in Wild Rice
Source: Plants (Basel). 2022 Apr 3;11(7):977. doi: 10.3390/plants11070977 (PMC9002458; doi:10.3390/plants11070977)
Supplement: Supplementary file 1 [file plants-11-00977-s001.zip › Table S1. Statistical analysis of the sequencing data.pdf]

Table S1. Statistical analysis of sequencing data.

Samples	Raw reads	Length<18	Length>30	Clean reads	Q30[a](%)
9311-0 h-1	26,126861	2411375	10011067	13,704419	97.14
9311-0 h-2	29,405783	5169800	6973103	17,262880	97.25
9311-0 h-3	19,871273	1933486	6110022	11,827765	97.65
9311-96 h-1	22,842008	2812559	8321291	11,708158	96.08
9311-96 h-2	23,650069	5421798	6502108	11,726163	97.91
9311-96 h-3	22,755398	4702726	7588022	10,464650	97.30
DC90-0 h-1	24,160870	2634669	9570465	11,955736	97.74
DC90-0 h-2	20,081098	2116326	7043589	10,921183	97.84
DC90-0 h-3	21,361852	2424128	8009649	10,928075	97.71
DC90-96 h-1	21,811625	3922384	6213888	11,675353	97.06
DC90-96 h-2	20,129922	3585454	5176247	11,368221	97.84
DC90-96 h-3	21,598987	5262119	4596198	11,740670	97.98

Notes: The superscript a represents percentage of bases with mass value greater than or equal to 30.
