# Supplementary figures and images for: An Integration of MicroRNA and Transcriptome Sequencing Analysis Reveal Regulatory Roles of miRNAs in Response to Chilling Stress in Wild Rice
Source: Plants (Basel). 2022 Apr 3;11(7):977. doi: 10.3390/plants11070977 (PMC9002458; doi:10.3390/plants11070977)

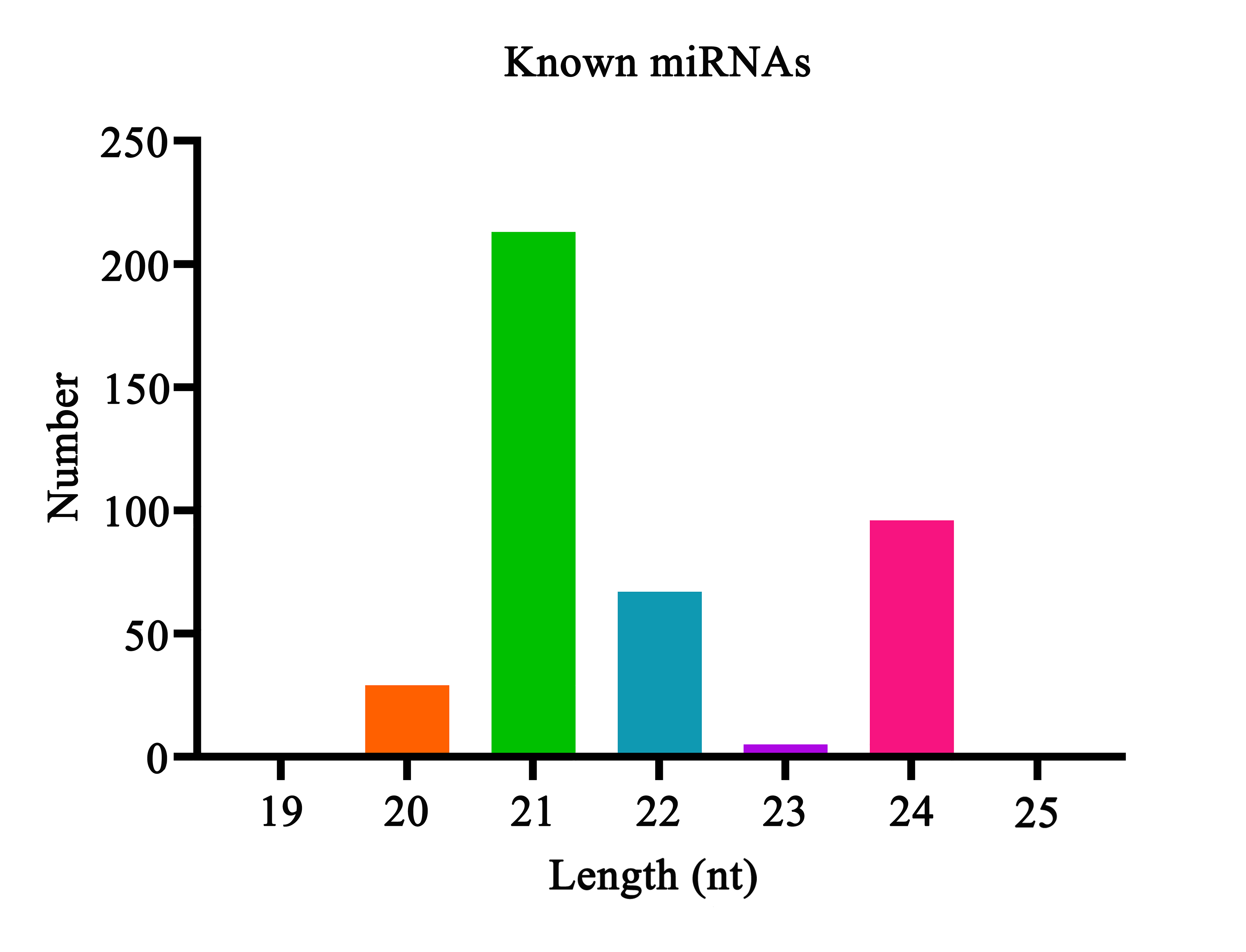

Supplement: Supplementary file 1 [file plants-11-00977-s001.zip › Figure S2.tif]

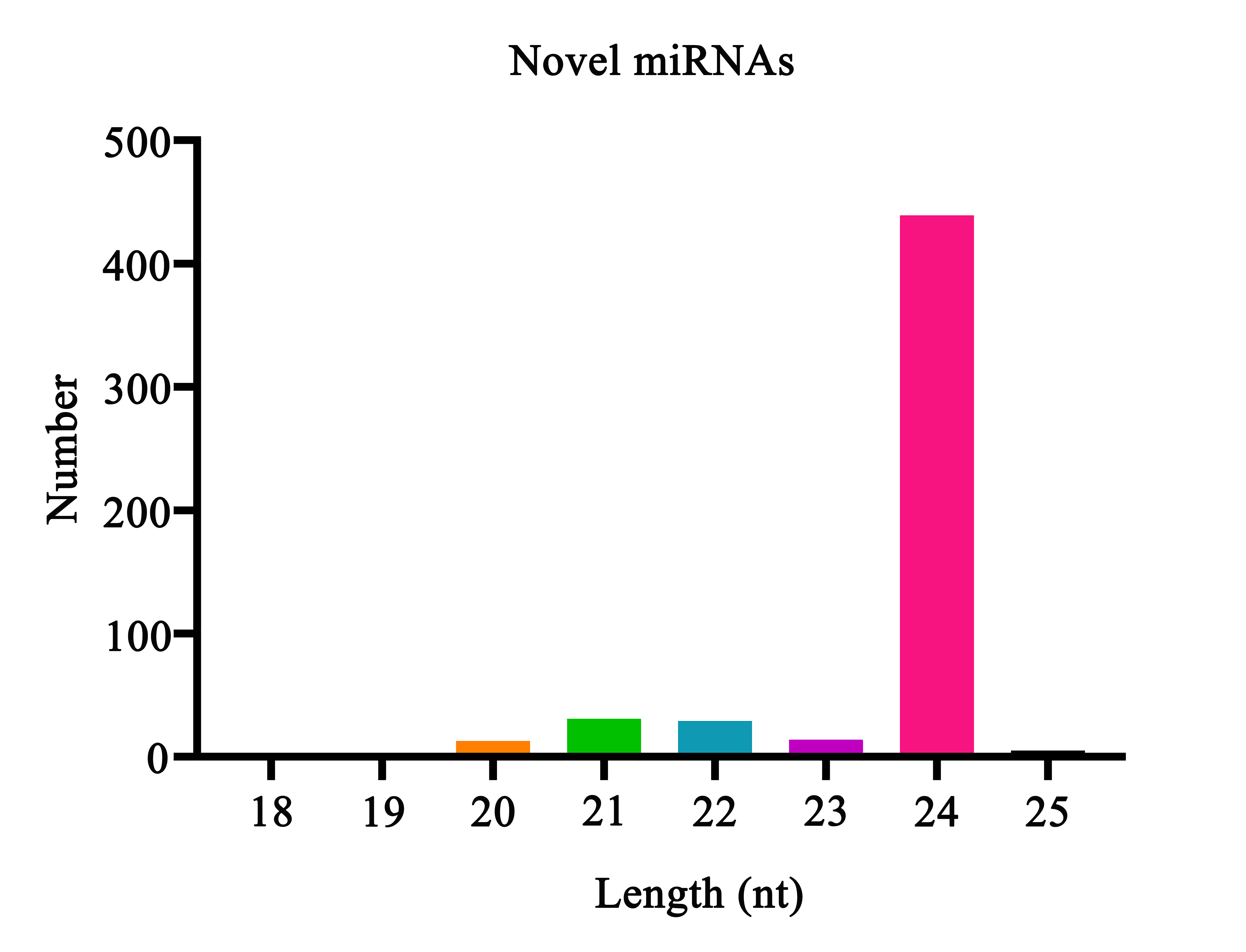

Supplement: Supplementary file 1 [file plants-11-00977-s001.zip › Figure S3.tif]
